# First Census of Patients with Hereditary Angioedema in the Canary Islands

**DOI:** 10.3390/jcm10204711

**Published:** 2021-10-14

**Authors:** Alejandro Mendoza-Alvarez, Itahisa Marcelino-Rodriguez, Lourdes Almeida-Quintana, Elena Martin-Fernandez, Dara Martinez-Beltran, Zulay Almeida-Sanchez, David Cruz-Niesvara, Guacimara Hernández-Santana, Jose C. Garcia-Robaina, Carlos Flores, Ariel Callero

**Affiliations:** 1 Research Unit, Hospital Universitario Nuestra Señora de Candelaria, Universidad de La Laguna, 38200 Santa Cruz de Tenerife, Spain; amendoal@ull.edu.es (A.M.-A.); itahisa@gmail.com (I.M.-R.); cflores@ull.edu.es (C.F.); 2 Allergy Service, Hospital Universitario de Gran Canaria Dr. Negrín, 35010 Las Palmas, Spain; lalmqui@gmail.com; 3 Allergy Service, Hospital General Dr. Molina Orosa, 35500 Las Palmas, Spain; elenamartinfernandez@hotmail.com; 4 Allergy Service, Hospital Universitario Insular-Materno Infantil, 35016 Las Palmas, Spain; daritamb@hotmail.com; 5 Allergy Service, Hospital General de La Palma, 38713 Santa Cruz de Tenerife, Spain; zulay85@hotmail.com; 6 Allergy Service, Hospital General de Fuerteventura Virgen de la Peña, 35600 Las Palmas, Spain; niesvaara888@yahoo.es; 7 Allergy Service, Hospital Nuestra Señora de Guadalupe, 38010 Santa Cruz de Tenerife, Spain; guacim1@hotmail.com; 8 Allergy Service, Hospital Universitario Nuestra Señora de Candelaria, Universidad de La Laguna, 38200 Santa Cruz de Tenerife, Spain; jgarrob@gobiernodecanarias.org; 9 Genomics Division, Instituto Tecnológico y de Energías Renovables, 38600 Santa Cruz de Tenerife, Spain; 10 CIBER de Enfermedades Respiratorias, Instituto de Salud Carlos III, 28029 Madrid, Spain

**Keywords:** hereditary angioedema, C1 esterase inhibitor, clinical diagnosis, diagnosis delay, descriptive study

## Abstract

Hereditary angioedema (HAE) is a rare genetic condition whose main symptoms are recurrent swelling in the skin, mucosa, and internal organs. Recent studies suggested that the regulation of the inflammatory response and the complement cascade are two of the pathways significantly enriched in the Canary Islands, Spain. Here, we describe the first HAE patient series in this region. Forty-one patients (33 F, 8 M) and nine healthy relatives belonging to twenty-nine families were recruited for this study, obtaining their clinical and demographic features using a data collection form, as well as blood samples for biochemical analysis. The mean age of patients was 36.8 years (ranging from 4 to 72 years). Positive family history of HAE was reported in 13 patients (32.5%), and a mean diagnosis delay of 7.9 (±12.5) years was estimated, ranging from months to 50 years. Cutaneous edema was the most common symptom (53.6%), while airway symptoms was present in 11 patients. Prophylactic treatment was indicated for 23 patients, while 14 also require on-demand rescue treatment. We estimate a minimum prevalence of 1.25:100,000 for HAE due to C1-INH deficiency or dysfunction in the Canary Islands, which is higher than the estimates for mainland Spanish populations. HAE continues to be a disease poorly recognized by health care professionals due to its confusing symptoms, leading to longer diagnosis delay. Altogether, the evidence reinforces the need for a rapid and accurate diagnosis and precision medicine-based studies to improve the patient’s quality of life.

## 1. Introduction

Hereditary angioedema (HAE) is a rare genetic disease whose symptoms are recurrent swelling (edema) affecting the skin, internal organs, mucosa or the upper airways [1,2]. The symptoms are caused by dysfunction of the C1 esterase inhibitor (C1-INH) or dysregulation of the kinin cascade, leading to bradykinin release and resulting in HAE attacks. Bradykinin is a vasoactive peptide and the main activator of the B2R receptor located in endothelial cells [3], whose activation leads to increased vascular permeability and edema, causing the HAE symptoms [4]. HAE attacks can turn into a life-threatening episode if the edema develops in the laryngeal tract, which leads to the obstruction of the upper respiratory airways [5]. HAE attacks are unpredictable and often curses with triggers such as mental stress [6], contraception hormones [7], infections [8], injury/surgery interventions [9], and weather changes [6], among others. Its prevalence has been estimated over 1:50,000 worldwide [10] and is reported in all ethnic groups [11].

This genetic condition is typically divided in two main groups attending to C1 esterase inhibitor (C1-INH) plasmatic levels. The most frequent form of HAE is caused by decreased levels of C1-INH (HAE-C1-INH) [11,12], where 85% of cases are explained due to lower C1-INH plasma levels (HAE Type I). The rest of cases are explained by normal levels of non-functional C1-INH protein (HAE Type II). A first national registry of HAE patients due to C1-INH deficiency or dysfunction in Spain was conducted by Roche et al., reporting a minimum prevalence of 1.09:100,000 [13]. In the other hand, there is another diagnostic group that show normal levels of functional C1-INH protein (HAE-nC1-INH), since the causal variants affect another point of the pathway [11]. The first genetic studies in HAE families with normal levels of C1-INH detected a mutation in the exon 9 of *F12* [14]. FXII activation increases the bradykinin accumulation, driving to increased vascular permeability. Surprisingly, only the 20% of patients with HAE-nC1-INH in Europe have causal variants in the *F12* gene [15]. In this context, the recent decreased cost of massive DNA sequencing allowed the first whole-exome sequencing (WES) studies to be completed in 2018, which detected variants affecting function in HAE-nC1-INH patients in the angiopoietin (*ANGPT1*) and plasminogen (*PLG*) genes [16,17]. More recently, Bork et al. identified variants in the kininogen 1 (*KNG1*) gene as another cause of HAE [18]. In the last year, two more genes have been associated with HAE-nC1-INH using next generation sequencing technologies, the myoferlin (*MYOF*) and heparan sulfate 3-O-sulfotransferase 6 (HS3ST6) genes [18]. Despite this, precise estimates of the prevalence of this latter subtype among HAE patients remains unknown.

Because of its nonspecific signs, HAE is poorly recognized in the clinical field, resulting in delayed diagnoses and undertreatment. An undiagnosed HAE is at higher risk of morbidity and mortality compared to those that had a diagnosis, especially if attacks affect the airways [19]. However, advances in testing procedures and disease recognition do not improve the diagnosis of HAE, which still presents a considerable challenge for physicians [20]. Currently, genetic testing is not frequently applied for the HAE clinical diagnosis routine in many health care systems, including the Canary Islands (Spain). However, updated HAE management guidelines recommend a genetic-based diagnosis to increase the diagnostic yield and reduce the diagnosis delay in those patients with HAE Type II and HAE-nC1-INH, as well in those cases with highly suspicious of HAE Type I diagnosis and negative family history [11,21]. At this moment, more than 450 HAE-C1-INH disease causing variants to have been reported in *SERPING1* gene, which encodes for C1-INH protein [11,22,23,24]. Until 2018, only two genes were associated with HAE, which is why clinicians continue to make HAE diagnosis based on plasmatic determinations of reported biomarkers. Furthermore, the debilitating nature of HAE attacks makes early diagnosis as a critical achievement to establish the effective treatment for short/long-term prophylaxis [25].

Due to the clinical importance of establishing an accurate diagnosis and prescribe the required treatment to HAE patients, several countries such as Denmark [26], Saudi Arabia [27], Turkey [28], Japan [29], Portugal [30], Greece [31], Norway [32], Switzerland [33], Croatia [34], China [35], Romania [36], Austria [37], Puerto Rico [38], and New Zealand [39], among others, are designing their own patient registry in order to investigate the underlying causes in HAE development, create protocols and facilities that helps clinicians to manage and diagnose the patients, improving the diagnosis delay.

For this reason, we present the first patient registry of Hereditary Angioedema on the Canary Islands with the main aim of analyzing the characteristics of HAE patient and establishing the grounds of precision medicine. This will help to set a biobank for subsequent analysis to improve the clinical and genetic diagnosis.

## 2. Materials and Methods

### 2.1. Study Design and Patient Recruitment

The patients included in this cross-sectional study were recruited by the Allergy services from main hospitals from the Canary Islands (Hospital Universitario Nuestra Señora de Candelaria, Tenerife; Hospital Universitario Dr. Negrín, Gran Canaria; Hospital General Dr. Molina Orosa, Lanzarote; Hospital Universitario Insular-Materno Infantil, Gran Canaria, Hospital General de La Palma, La Palma; Hospital Nuestra Señora de Guadalupe, La Gomera; and Hospital General de Fuerteventura Virgen de la Peña, Fuertenventura), between January 2015 and March 2021. We included the cases that had compatible clinical history of angioedema attacks without urticaria, and a differential diagnosis by blood molecular assays. We excluded patients using medication that were known to trigger angioedema attacks (i.e., angiotensin-converting enzyme inhibitors) and patients suffering several pathologies that potentially cause angioedema attacks (e.g., hepatitis, HIV, hepatic/renal disorders, immunological deficiencies, and infections due to Helicobacter pylori).

HAE diagnosis was exclusively based on symptoms and biochemical determinations, according to the international WAO/EAACI guidelines for the management of HAE [39]. Clinical data were obtained from patients through a specifically designed form, including age at first symptoms, date of diagnosis, frequency of HAE attacks and localization in the body, plasmatic C4 and C1-INH determinations, positive genetic testing, among others. As part of the clinical diagnosis routine, Sanger sequencing to detect the causal variant were carried out by the genetic analysis laboratories of each main hospital only for HAE-nC1-INH patients due to the unavailability of kits for *SERPING1* screening in the moment of patient recruitment. A severity score for this disease was adapted from Ferraro et al., to quantify the severity course of the disease for each patient [40]. As is described in this study: “episodes of angioedema were classified according to average frequency and intensity of symptoms since onset of disease; to improve accuracy, mean values were obtained for the past 5 years for patients with long-term histories. Frequency of symptoms was quantitated as follows: more than one episode a month, three points; between 6 and 11 episodes a year, two points; <6 episodes a year, one point; and no symptoms of angioedema, zero point. Intensity of symptoms was classified as: presence of discomfort but no disruption of daily activity, 2 points; discomfort reducing normal daily activity, 4 points; and inability to work or per-form daily activity and/or necessity of hospital care, 5 points”. Computing all points for the severity course of the disease, recruited patients can be classified in four groups: asymptomatic (0 point), mild (≤4 points), moderate (5–6 points), or severe (≥7 points).

All biological samples and clinical information collected from donors were centralized and stored at the Research Unit of Hospital Universitario Nuestra Señora de Candelaria (HUNSC). The study was approved by the HUNSC Ethics Committee (PI 57–17) and written informed consent was obtained from the patients.

### 2.2. Molecular Diagnosis

C4 and C1-INH plasmatic levels were determined by nephelometry (N Antisera to Human Coagulation Factors and C1 Inhibitor, BN II: Siemens Healthcare Diagnostics, Marburg, Germany), while the C1-INH activity determination was performed by functional chromogenic assay (Berichrom C1-inhibitor, Siemens Healthcare Diagnostics, Marburg, Germany). Clinical plasma determinations of C4 and C1-INH were performed by clinical diagnosis laboratories of each associated hospital, while C1-INH function assay was carried out by Reference Laboratory (Barcelona, Spain). As expected, HAE-nC1-INH patients were also present in the series, here described as patients with normal or slightly decreased C1-INH levels and function.

To exclude patients with acquired angioedema (AAE), we determined the C1q and Anti-C1q autoantibodies levels in those patients with C1-INH deficiency and without referred family history, with onset at late ages, or association with lymphoproliferative tumors.

### 2.3. Statistical Analysis

Variables measured with a normal scale are reported as summary statistics, that is, the mean (±SD), and nominal variables are reported as absolute and relative frequencies (%) for each category. The Student’s t-test was used for bivariate comparisons of continuous variables, and continuous and categorical variables were compared with analysis of variance (ANOVA). Chi-squared tests were used to compare proportions between emergency room visits and the diagnosis delay. Statistical analyses were conducted with SPSS v.21 software (IBM Corp., New York, NY, USA).

## 3. Results

### 3.1. Demographic and Clinical Patient Outcomes

A total of 41 affected patients and nine healthy related controls belonging to 28 families were recruited for this study (Table 1). Eight male patients (19.5%) and 33 females (80.5%) were present in this series. The patients were aged between 4 and 72 years (mean: 36.8 ± 16.9 years) and positive family history for HAE attacks was reported in 13 of them (32.5%). According to C1q and anti-C1q autoantibodies, we only detected one patient with C1q deficiency, and that patient was excluded from the current study as it was deemed to be a AAE diagnosis. All types of HAE are present in the series, being HAE-C1-INH the most abundant, with twenty-two individuals diagnosed for HAE Type I (53.7%) and five patients for HAE Type II (12.2%). The reminder 14 individuals (34.1%) were diagnosed as HAE-nC1-INH, which were mainly composed by females (92.8%). Despite the recommendations of the updated HAE management guidelines, genetic testing was not widely applied in the diagnosis routine at the different hospitals from the Canary Islands due to the unavailability of kits for *SERPING1* screening. Only negative genetic tests based on a targeted interrogation of p.Thr328Lys located in *F12* gene variant were obtained from seven HAE-nC1-INH patients with positive familiar history.

Severity scores were only available for 40 recruited patients due to loss of clinical follow-up in one patient as a reason of change of address to another region. According to the severity scale established for this study, the most frequent group of patients has a mild severity (*n* = 20), while the less frequent patient groups had moderate (*n* = 10) and severe (*n* = 5) courses of the disease. The rest of patients were classified as asymptomatic (*n* = 5) due to the previously prescription of HAE treatment. Severity scores were not correlated with the type of HAE (*p* = 0.551; Levene’s test).

**Figure 1:**
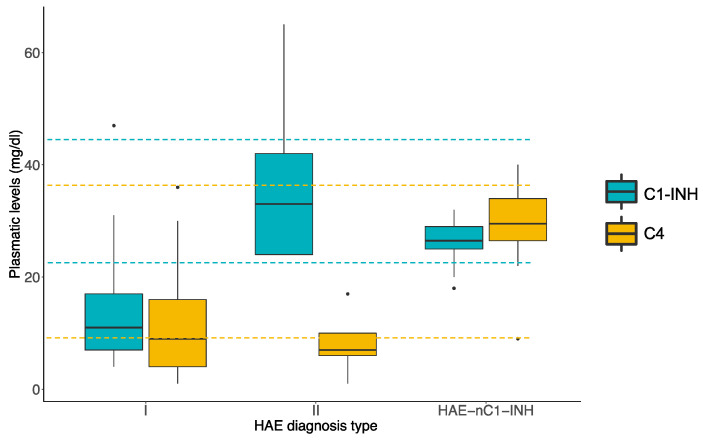
Plasmatic concentration of C1-INH and C4 among the different diagnosis type of HAE with reference values for each protein. The dotted lines indicate the upper and lower reference values for the corresponding proteins (indicated by colors) and black dots represents outliers determinations of plasmatic protein concentration.

The C1-INH plasmatic levels were lower for HAE Type I than for Type II patients (Figure 1), in agreement with data published elsewhere (*p* = 7.99 × 10^−3^; one-way ANOVA) [5,41,42]. In addition, the HAE-nC1-INH patients showed the highest C1-INH plasmatic concentration (*p* = 1.42 × 10^−3^; one-way ANOVA), while C4 values were midway between those of HAE Type I and Type II patients (*p* = 5.85 × 10^−6^; one-way ANOVA). These results agree with HAE diagnosis guidelines [5,39,41].

### 3.2. Treatment Indication

Regarding to the treatment prescriptions, 23 patients required a specific treatment for long-term prophylaxis (Table 2). Here, the attenuated androgens were the most frequent prescribed treatment (*n* = 16), where 11 patients were indicated for stanozolol and five for danazol. In the rest of patients, antifibrinolytics were prescribed only when C1-INH concentrate was not available for self-administration at home, and androgens were contraindicated (*n* = 7) [39]. In addition, a rescue treatment at home for spontaneous HAE attacks were indicated for 14 of the patients. Icatibant acetate was the recommended drug for emergency at-home situations. Finally, all patients were advised of the availability of purified C1 inhibitor in the emergency services in case of angioedema attacks.

## 4. Discussion

Here, we present the first registry of patients diagnosed with HAE in the Canary Islands, Spain. With this study, we describe their main clinical characteristics and aimed to configure a cohort for subsequent precision medicine studies.

In Spain, the estimated prevalence of individuals affected by HAE Type I and Type II is 1.09:100,000 [13], being slightly lower than the prevalence obtained in other European studies, estimated around 1:50,000 [43]. Nevertheless, in the Canary Islands alone, we found 41 affected individuals, obtaining a prevalence of 1.90:100,000, which is higher than that reported for overall Spain. However, this figure has been estimated including all HAE cases regardless of type. Attending only to the patients with HAE due to C1-INH deficiency or dysfunction (*n* = 23), the estimated prevalence is 1.25:100,000, which is still higher than the overall estimate for mainland Spanish populations. This could be explained by their isolation and the recent demographic history of the Canary Islands populations. In agreement with this, we have found in previously genetic studies the regulation of inflammatory response and the complement cascade as two significantly enriched pathways [44]. This finding, in association with an estimated higher prevalence of HAE in the Canary Islands, may add to the underlying genetic factors involved in angioedema that have not been identified to date. Besides, the prevalence for HAE-nC1-INH remains unknown in Spain.

Although HAE is a disease with an autosomal dominant pattern, our patient series was mainly composed by females (33/41). Thus, a sexual disbalance is present in the cohort. This could be explained by the protective role of male hormones, as it was observed in other HAE patient study series [45]. Indeed, the high predominance of affected women may also be related to hormones, being the estrogens one of the main triggers of attacks in HAE [46,47]. However, we found that symptomatic females started to develop their symptoms sometime after the puberty period on average (25.4 ± 12.8 years), the period when most women affected by HAE debuts with symptoms.

Like in other rare diseases, the determination of the correct clinical diagnosis helps the patients to reduce their level of uncertainty and stress caused by the symptoms. This is especially important in the context of HAE, because one of the most powerful triggers is the psychological stress [6]. This situation contributes to more frequent attacks, requiring more visits to the emergency room visits, or if the patient has an established HAE diagnosis, requiring the rescue treatment [48]. However, HAE remains a clinical entity not known by most healthcare professionals, especially in the emergency departments, which has been demonstrated by the long-time interval between the onset of symptoms and diagnostic confirmation [45]. Besides, HAE is a rare genetic condition with a very high morbidity and mortality rate in patients who do not have an indicated prophylaxis or rescue treatment, leading to a high cost to the healthcare system mainly caused by the frequent absenteeism from work and the loss of personal autonomy [43]. In this context, having a positive familiar history of HAE could contribute to reduce the diagnosis delay. However, the national surveys carried out in Italy determined that only 45% of the patients had a positive family history, which could be explained by the underdiagnosis of relatives [49].

Several studies have focused on HAE penetrance, determining in some cases a low penetrance that would explain why individuals with plasma levels of C1-INH and C4 and C1-INH activity below the reference values remain asymptomatic [26,50]. In fact, three asymptomatic relatives recruited for this study presented plasmatic levels for these proteins that would lead to a diagnosis of HAE. Indeed, this situation is somewhat beneficial, due to the helpful experience of their relatives regarding the management of attacks and the presence of on-demand rescue treatment at home, allowing an anticipation by the clinical team and a rapid diagnosis of HAE. In this context, symptomatic individuals have shown a higher age at onset compared to other epidemiological studies (23.9 years), where most of the patients debut at pediatric age [37,45,51].

Current guidelines for the management of patients with HAE recommend that treatments must be based mainly on long-term prophylaxis and on-demand rescue options, with a short-term prophylaxis required before surgical procedures [21,39]. In our series, 40.3% of symptomatic patients have an indicated treatment for long-term prophylaxis and 34.1% already have an on-demand rescue treatment at home (icatibant acetate). In addition, the international WAO/EAACI guidelines recommends reassessing the long-term prophylaxis treatment in each follow-up visit, giving more priority to on-demand treatment to reduce the side effects of long-term prophylaxis treatment and improving the quality-of-life patients with HAE [39].

Finally, based on biobanked samples from this series, we aim to cover the possibility to perform genomic studies to determine the underlying genetic causes and, ideally, identify new genetic causes or disease modifiers. In this context, we have recently designed HADA, a user-friendly tool that incorporates a genomic database of 451 previously reported causal variants affecting function [11] in genes that are known to underlie HAE (http://hada.hpc.iter.es/; accessed on 7 September 2021) [22]. This tool and the underlying database are under constant revision (latest made on 19 July 2021: https://github.com/genomicsITER/HADA/blob/master/CHANGELOG.md; accessed on 19 July 2021) to continue allowing the professionals to assist in the prioritization genetic variants for the accurate diagnosis of this rare disease. With these other genomic assessments, we aim to contribute to reduce the diagnosis delay and improve the quality of the patient life.

## 5. Conclusions

To our knowledge, this is the first HAE study conducted in the Canary Islands, a population showing one of the highest prevalence of allergic diseases in Spain, which could be partially explained by the population isolation and the particular genetic background. The establishment of this patient registry and the obtention of the clinical data, as well as the availability their biological material, will allow us to propose genomic studies to determine the underlying genetic causes of HAE in the Canary Islands.

## Figures and Tables

**Table 1:** Clinical features of the patients and healthy relatives recruited in this study.

	Findings
Recruited number	41
Age, years (mean ± SD)	37.1 (±17.0)
Sex	
Male	8 (20%)
Female	33 (80%)
Clinical diagnosis	
HAE Type I	22 (53.7%)
HAE Type II	5 (12.2%)
HAE-nC1-INH	14 (34.1%)
Positive family history	13 (32.5%)
Age at first symptom (mean ± SD)	23.9 (±13.9)
Age at diagnosis (mean ± SD)	31.8 (±16.6)
Diagnostic delay (mean ± SD)	8.1 (±12.6)
Pretreatment period	
Emergency room visits [median/IQR (25–75)]	1.0/0.0–2.0
Required hospitalization [median/IQR (25–75)]	0.0/0.0–0.0
Number of episodes by year [median/IQR (25–75)]	5.0/1.5–5.0
Airways [median/IQR (25–75)]	0.0/0.0–1.0
Abdominal [median/IQR (25–75)]	0.0/0.0–4.7
Cutaneous [median/IQR (25–75)]	2.0/0.0–5.0
Facials [median/IQR (25–75)]	1.0/0.0–4.0
Post-treatment period	
Emergency room visits [median/IQR (25–75)]	0.0/0.0–0.5
Required hospitalization [median/IQR (25–75)]	0.0/0.0–0.0
Number of episodes by year [median/IQR (25–75)]	2.0/0.0–5.0
Airways [median/IQR (25–75)]	0.0/0.0–0.0
Abdominal [median/IQR (25–75)]	0.0/0.0–1.0
Cutaneous [median/IQR (25–75)]	0.0/0.0–3.7
Facials [median/IQR (25–75)]	0.0/0.0–1.0
Score (*n* = 39)	
Asymptomatic (%)	5 (12.5%)
Mild (%)	20 (50.0%)
Moderate (%)	10 (25.0%)
Severe (%)	5 (12.5%)

HAE: hereditary angioedema; SD: standard deviation; IQR: interquartile range.

**Table 2:** Treatment for hereditary angioedema patients.

	*n*
No treatment	20 (46.5%)
Attenuated androgens	
Danazol	5 (11.6%)
Estanozolol	11 (25.6%)
Antifibrinolytic (tranexamic acid)	7 (16.3%)
Rescue treatment (icatibant acetate)	14 (32.6%)

## Data Availability

The data presented in this study are available on request from the corresponding author. The data are not publicly available due to the restrictions established by the HUNSC Ethics Committee (PI 57-17).
